# Reply to Nepomuceno et al.: A renewed call for detailed social and demographic COVID-19 data from all countries

**DOI:** 10.1073/pnas.2009408117

**Published:** 2020-06-23

**Authors:** Jennifer Beam Dowd, Liliana Andriano, David M. Brazel, Valentina Rotondi, Per Block, Xuejie Ding, Melinda C. Mills

**Affiliations:** ^a^Leverhulme Centre for Demographic Science, Nuffield College, University of Oxford, Oxford OX1 3UQ, United Kingdom

We thank Nepomuceno et al. (1) for their thoughtful comments and welcome this extension. We strongly agree that in addition to age and sex, additional demographic factors such as the prevalence of comorbidities and broader social determinants are crucial for understanding the impact of coronavirus disease 2019 (COVID-19) on mortality within and across countries. Reiterating our conclusion, we note that the benefit of younger age structures in lower-income countries may be partially offset by weak health systems and a higher prevalence of HIV, tuberculosis, and other chronic conditions (2). In the midst of shockingly high mortality in Italy, our aim was to highlight the potential impact of population age structure on COVID-19 mortality, holding other factors constant. Naturally, the real world reflects more complex dynamics, but the strong association between age and COVID-19 mortality remains. Since disaggregated and individual-level data to understand these multiple risks remain scarce, we renew our call and join others in demanding the timely release of detailed social and demographic COVID-19 data from all countries (3).

We disagree with the authors’ interpretation that we “implicitly assumed the age prevalence of underlying comorbidities is similar in Italy, Brazil, and Nigeria” in our illustrations of variations in total deaths dependent on population age structure. Our direct comparison was in fact between Brazil and Nigeria, chosen as two countries with similar population sizes but different age structures. Although we applied the case fatality rates (CFRs) of Italy for our illustration, the result that age structure predicts total fatalities across countries depended not on specific rates but rather on the strong age gradient in mortality, which would have remained similar using CFRs from China, for example.

The authors raise the important point that biological risk reflects factors beyond chronological age, and that lower- and middle-income countries now face a great unknown of how underlying health and social vulnerabilities will interact with COVID-19. Indeed, 76% of official COVID-19 deaths in Brazil have occurred at ages 60 y and older compared to 95% for Italy (4). While these figures do not allow direct comparison of CFRs due to the age distribution of cases and ascertainment of both cases and deaths (5), they suggest that less-healthy countries may see a shift toward a younger age distribution of COVID-19 deaths relative to wealthier and healthier countries, consistent with high rates of comorbidities such as diabetes and hypertension in Latin American countries (6). Emerging data from the United States and United Kingdom also show that some ethnic minorities and socioeconomically disadvantaged populations have a higher COVID-19 mortality risk (7–9). Accelerated biological aging observed with social disadvantage (10) will undoubtedly shape the imprint of COVID-19 across the world. As of this writing, young populations in Africa and South Asia remain less severely impacted (11), maintaining hopes of a protective effect of youth on COVID-19 mortality, even in the face of broader risks.
